# Creutzfeldt-Jakob Disease Mortality in Japan, 1979-2004: Analysis of National Death Certificate Data

**DOI:** 10.2188/jea.17.133

**Published:** 2007-07-18

**Authors:** Yuriko Doi, Tetsuji Yokoyama, Miyoshi Sakai, Yosikazu Nakamura

**Affiliations:** 1 Department of Epidemiology, National Institute of Public Health.; 2 Department of Technology Assessment and Biostatistics, National Institute of Public Health.; 3 Department of Public Health, Jichi Medical University.

**Keywords:** Creutzfeldt-Jakob Syndrome, Regression Analysis, Mortality, Death Certificate, Japan

## Abstract

**BACKGROUND:**

Trend of the mortality rate of Creutzfeldt-Jakob disease (CJD) in Japan is still unclear. This study aimed to estimate annual crude mortality rates due to CJD and examine the CJD mortality trend in Japan during the period of 1979-2004.

**METHODS:**

National death certificate data on CJD were used (CJD coded as 046.1 for ICD-9 and A81.0 for ICD-10). Trends in age-standardized mortality rates for CJD were examined by using time series analyses including the Joinpoint regression analysis.

**RESULTS:**

A total of 1,966 deaths (862 males and 1,104 females) were identified with CJD coded as the underlying-cause-of-death. The annual number of deaths and crude mortality rates peaked in 2004 at 163 (66 for males and 97 for females) deaths and 1.28 (1.06 for males and 1.48 for females) deaths per million population per year, respectively. The age-specific mortality rates rapidly increased with age between 50 and 74 years, especially among females, and sharply declined at 80+ years. Throughout the observed period, there were no significant change points, and the annual percentage changes (95% confidence intervals) were +3.09 (2.18-4.02) % for males and +3.90 (2.98-4.83) % and females. The total number of CJD deaths under 50 years of age was 131, and there was found no increase in the annual number of deaths for the past few years in this age group.

**CONCLUSION:**

CJD mortality in trend data based on death certificates has significantly increased in Japan during the period of 1979-2004.

Creutzfeldt-Jakob disease (CJD), the most common human prion disease or transmissible spongiform encephalopathy, is a rapidly progressive neurodegenerative disorder with a fatal outcome. It is divided into four types: sporadic, familial, iatrogenic, and variant CJD.^1^ The disease has received academic as well as public attention in Japan because of iatrogenic CJD (iCJD) transmitted through cadaveric dura grafts ^2^^-^^5^ and variant CJD (vCJD) suspected to be associated with bovine spongiform encephalopathy (BSE).^6^^,^^7^ Contaminated cadaveric dura grafts with the CJD agents were used for neurosurgery in Japan during the period of 1978-1991.^8^ Miyashita reported a case with CJD, in 1991, who had received the contaminated cadaveric dural material 33 months before the onset of the disease.^9^ In 2006, Yamada reported the first Japanese case of definite vCJD, who were affected at the age of 48 in 2001.^7^

A nationwide hospital-based survey on CJD, conducted in 1996, previously pointed out the increase of incidence and mortality rates of the disease during the period of 1985-1995 ^10^ but after then the trend of frequency of the disease in Japan has not been observed. It is important to estimate the mortality rates of CJD, and thus we examined the CJD mortality trends in Japan for the extended period of 1978-2004.

## METHODS

### Data

Cause-of-death classifications are based on the International Classification of Diseases, Ninth (ICD-9)^11^ and Tenth Revisions (ICD-10).^12^ These documents designate CJD deaths by codes 46.1 and 331.5, and A81.0 and F02.1, respectively. In Japan, 46.1 and A81.0 are valid codes for the underlying cause-of-death for CJD during the periods of ICD-9 (from 1979 through 1994) and ICD-10 (from 1995 and thereafter). Although the grafting of the contaminated cadaveric dural material had begun in 1978, we had to skip 1978 due to unavailability of coding for CJD in the ICD-8 (from 1968 through 1978).^13^

In Japan, death certificates are systematically stored on magnetic tape data files by the Ministry of Health, Labour and Welfare. These certificates are filled in by medical doctors at hospitals or clinics, and are changed into computerized files at the Ministry. We used the mortality data (1979-2004) on CJD based on death certificates derived from the computer tapes with the permission of the Ministry. The data files used contained the codes for the underlying cause-of-death for CJD as well as basic information coded for sex, age, date and place at death, date of birth, household occupation, and place of residence where the deceased had lived. It did not contain an individual's name or residential address.

Population data were obtained from the 1975, 1980, 1985, 1990, 1995, and 2000 censuses. For each year between the censuses, population estimates were interpolated by using a linear model. The 2005 census data were not available at the time of this analysis so that the population estimates for 2001-2004 were used, being provided by the Statistics Bureau, Ministry of Internal Affairs and Communications.^14^

### Statistical Analyses

The total number of deaths due to CJD from 1979 through 2004 was counted, and age-specific mortality rates were calculated according to 5-year age interval groups. The annual number of deaths from CJD was counted for each year during the observed period. In addition, the annual number of CJD deaths under 50 years of age was counted over this period. Because vCJD, which emerged in the United Kingdom in 1994, has the distinct clinical course features; the median age at death was 28 years, and most of the cases died at an age under 50 years.^6^^, ^^15^

CJD mortality rates in 1979-2004 were classified and obtained in two ways: (1) The annual crude mortality rates were calculated as the number of CJD deaths per million persons per year, on the basis of the Japanese populations for the respective years aforementioned; and (2) the annual age-standardized mortality rates were calculated by the direct method^16^ using the 1985 Japanese standard population.

Trends in age-standardized mortality rates for CJD were firstly examined graphically using a moving average technique, and then quadratic and cubic regression analyses were conducted to test thenon-linear trend. The trends were also analyzed by using a join-point regression model.^17^ The joinpoint regression technique is useful for delineating changes in trend data, especially when the number of change points is unknown. This method identifies the number of significant change points by performing a sequence of permutation tests of the null against alternative hypotheses to select the final model. The joinpoint regression analysis provides the estimated annual percentage change and the corresponding 95% confidence interval (CI). In addition, autocorrelation coefficients were calculated to examine the independency of residuals from the regression line.^18^

Statistical analyses were performed using SAS^®^ version 9.1.3 and SEER*^®^ Stat software, developed by the Surveillance, Epidemiology, and End Results Program of the National Cancer Institute of the USA, was employed for the joinpoint regression analysis.^19^

## RESULTS

**Figure 1.**
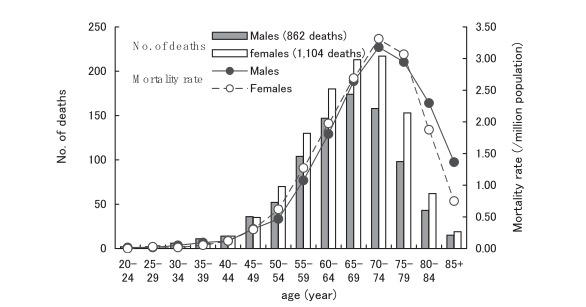
Number of deaths and mortality rates due to Creutzfeldt-Jakob disease by age, Japan, 1979-2004.

Table 1 shows the sociodemographic characteristics of the deceased from CJD. Of a total of 1,966 deaths (862 males and 1,104 females), 56.2% were females, and 75.2% were persons aged 60 years and older. The household occupation for half of the deaths was being unemployed. Most deaths (95.2%) occurred at hospitals. Figure 1 shows the number of deaths and mortality rates due to CJD by 5-year age groups. The age-specific mortality rates increased rapidly with age, particularly between 50 and 74 years of age, and declined sharply in persons older than 80 years of age. The rates were slightly higher in females than males for those aged 50-79 years. There were no CJD deaths under 20 years of age.

**Table 1.** Creutzfeldt-Jakob disease deaths by sex, age, household occupation, and location of death, Japan, 1979-2004.

Characteristics		n	%
Sex	Male	862	43.8
	Female	1,104	56.2

Age (year)	-19	0	0.0
	20-29	7	0.4
	30-39	25	1.3
	40-49	99	5.0
	50-59	356	18.1
	60-69	714	36.3
	70-79	626	31.8
	80+	139	7.1

Household occupation	Agriculture	162	8.2
	Self employed	199	10.1
	Employee I*	235	12.0
	Employee II^†^	220	11.2
	Other^‡^ /Unemployed	1,150	58.5

Location of death	Hospital ^§^	1,876	95.4
	Clinic ^§^	24	1.2
	Home	54	2.7
	Nursing Home/Other	12	0.6

* : Employee I indicates a full-time worker as manager, clerk, teacher, sales person, engineer or health professional.

† : Employee II is a full-time worker other than Employee I.

‡ : Other means a part-time or contingent worker.

§ : Hospitals and clinics have 20 or more and 19 or less inpatient beds, respectively.

**Figure 2.**
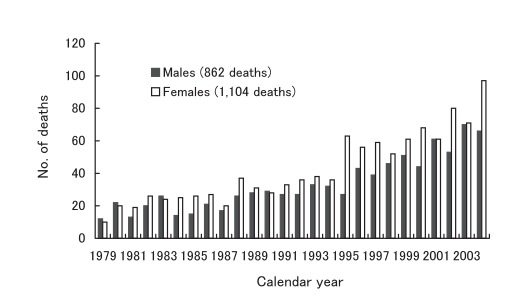
Annual number of deaths due to Creutzfeldt-Jakob disease, Japan, 1979-2004.

**Figure 3.**
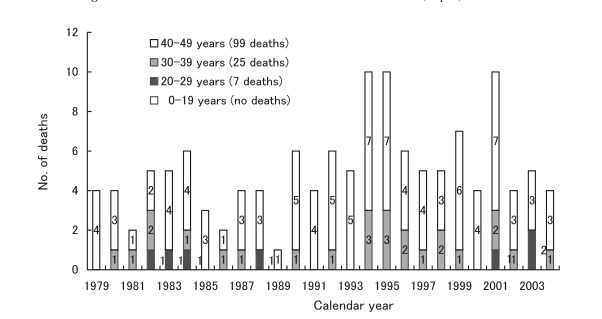
Annual number of deaths due to Creutzfeldt-Jakob disease under 50 years of age, Japan, 1979-2004.

There was a variation in the number of annual CJD deaths, from the minimum of 22 in 1979 to the maximum of 163 in 2004 (Figure 2). Figure 3 shows CJD deaths under 50 years of age. Of those, the highest annual records (10 deaths per year) occurred in 1994, 1995, and 2001. For the recent 3 consecutive years, however, the annual number of deaths slightly decreased in this age group (4 to 5 deaths per year).

**Figure 4.**
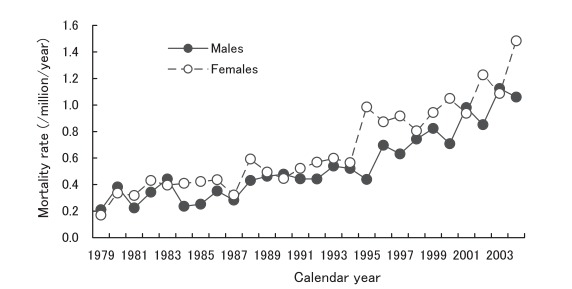
Annual crude mortality rates due to Creutzfeldt-Jakob disease, Japan, 1979-2004.

Figure 4 presents annual crude mortality rates due to CJD from 1979 through 2004. The rates ranged from 0.21 /million/year in 1979 to 1.12 in 2003 for males, and from 0.17 in 1979 to 1.48 in 2004 for females.

**Figure 5.**
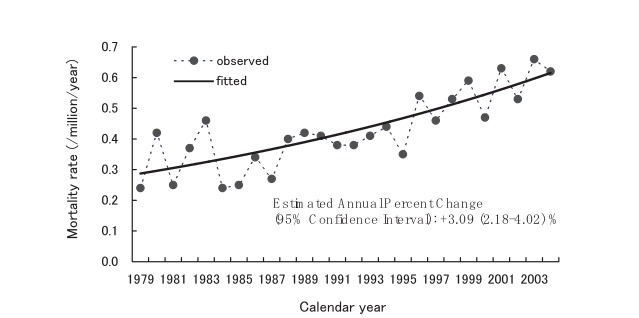
Trends in annual age-standardized mortality rates due to Creutzfeldt-Jakob disease among males, Japan, 1979-2004.

**Figure 6.**
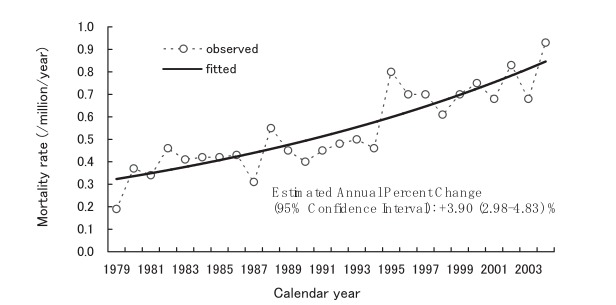
Trends in annual age-standardized mortality rates due to Creutzfeldt-Jakob disease among females, Japan, 1979-2004.

Figures 5 and 6 display the annual age-standardized mortality rates due to CJD for males and females, respectively. According to the joinpoint regression analyses, there were no significant change points in the trends from 1979 through 2004. For each trend, the corresponding figure presents a single linear regression line fitted with age-standardized mortality rates. The estimated annual percentage changes (95% CIs) were +3.09 (2.18-4.02) % for males and +3.90 (2.98-4.83) % for females.

The trends in the annual age-standardized mortality rates for CJD were examined graphically using a moving average technique, and confirmed that the increasing trends were approximately linear. In addition, quadratic and cubic regression analyses were conducted to test the non-linear trend, but it was not statistically significant. These findings supported the results obtained from joinpoint regression analyses that showed no change points during the observational period. Autocorrelation coefficients for residuals of the regression line were small and non-significant regardless of the time intervals between data points, indicating that the deviations of annual age-standardized mortality rates from the regression line were random noise.

## DISCUSSION

The present study identified the total number of 1,966 deaths (862 males and 1,104 females) due to CJD based on death certificate data in Japan during the period of 1979-2004. The number of deaths and crude mortality rates per year peaked in 2004 at 163 (66 for males and 97 for females) deaths and 1.28 (1.06 for males and 1.48 for females) deaths per million population, respectively. We discuss, first of all, whether or not CJD mortality has increased in Japan over the past 26 years. As demonstrated in this study, we found a significant linear increase in trends for age- standardized mortality rates from the disease, with +3-4% of annual percentage change, between 1979 and 2004. In interpreting the results, we should consider some factors that might contribute to a false increase in mortality, such as the change of ICD codes and the enhancement of case findings (e.g., physicians’ recognition of the disease, diagnostic tests, and quality of health care). No revolutionary new diagnostic test for CJD became available throughout the observational period. On the other hand, there were a few critical points of time to consider: in 1991, patients with CJD transmitted by cadaveric dura transplants were identified in Japan;^9^ in 1995, the ICD code for CJD was changed from 9th to 10th version in Japan; and in 1996, a new case of vCJD causally linked to BSE was reported from the United Kingdom.^6^ Without an abrupt rise of age-standardized mortality rates from CJD after these years for both sexes, however, it is unlikely that these events artificially affected the increase in CJD mortality.

Rather, it may be the true fact that in Japan our results reflect to a large extent a genuine increase in CJD. The number of iCJD cases may still increase even after the total ban on the practice of causal grafts.^5^^, ^^8^ Regarding sporadic CJD (sCJD), a recent report from the European Union's collective study on CJD suggests that the mortality rates from sCJD increased with time between 1993 and 2002.^20^ It is quite probable that this temporal increase of sCJD may also exist in Japan. The increase may have been accompanied to some extent by the improvement of physicians' diagnostic skills for CJD since 1997 when a manual for clinical practice on CJD was introduced in our country.^20^^, ^^21^

Consistent with the previous findings,^22^^-^^28^ the present study showed that the CJD mortality rates rapidly increased with age between 50 and 74 years, especially among females, and sharply declined at 80+ years of age, although the causal mechanism remains unexplained. These findings were comparable with those for patients with CJD reported to the Surveillance.^29^^, ^^30^

We focus hereafter our discussion on CJD deaths in young patients, younger than 50 years of age. The vCJD associated with BSE has a course and pathology distinct from sCJD: younger age at onset, prominence of psychiatric and sensory symptoms, and a long disease course.^6^^, ^^15^ Most of such cases died at an age under 50 years.^15^ In Japan, there has been only one case with definite vCJD, confirmed by the Japanese Creutzfeldt-Jakob Disease Surveillance Committee, who died at the age of 51 in December 2004, and had a history of visiting the UK, 1990, when the BSE outbreak was increasing there, but the causal association is unclear.^7^ As Bradley and Liberski pointed out,^31^ human populations are still exposed to epidemics of BSE, although the epidemics in different countries are at different stages: the UK, the rest of the EU, and North America and other countries including Japan have all reported BSE in native-born cattle. Until January 2007, a total of 198 patients with vCJD were identified around the world: 162 in the UK, 21 in France, 4 in Ireland, 3 in the USA, 2 in Netherlands, and 1 each in Canada, Italy, Portugal, Spain, Saudi Arabia, and Japan.^15^ Thus it is important for us to trace back the frequency of CJD deaths for this age group in the past, in terms of roughly estimating the background risk. We could not ascertain the clinical types of CJD deaths observed in our study because of the lack of such information. We assume, however, excluding a single case of vCJD, 57%, 29% and 14% for sporadic, iatrogenic and familial types of CJD among the deaths, respectively, from the findings reported to the Surveillance.^30^

We finally refer to the validity of the data used in our study, which were based on the underlying cause-of-death obtained from death certificates. Potential inaccuracies in death certificate data used for our study could not be ignored because autopsy findings and clinical information to confirm the diagnosis of CJD were not available (e.g., definite, probable, and possible; sporadic, familial, iatrogenic, and variant). However, the seriousness is not so great, considering the following points: (1) Over 90% of CJD patients die within a few years of the onset of symptoms;^15^^, ^^23^^, ^^24^^, ^^29^^, ^^30^^, ^^32^ (2) Diagnosis is ascertained at the end of the clinical course; and (3) 95.2% of CJD deaths observed in this study occurred at hospitals.

Despite these limitations, the national death certificate data can work as an efficient tool for monitoring CJD mortality because it covers all the deaths from CJD that have occurred throughout the country. In addition, an annual review of national CJD death certificate data has the possibility of providing incidence information as a surrogate for ongoing CJD surveillance in Japan, regarding a quite short period of time from the onset through death of this disease. This will be effective, especially for the detection of small clusters of deaths at an age under 50 years, of which the occurrence still remains low. So, the combined data of death certificate and surveillance information should be taken into account for monitoring morbidity and mortality of CJD in the future.

In conclusion, our present study suggests that CJD mortality based on death certificate data significantly increased in Japan during the period of 1979-2004.
